# The expression of glucocorticoid and mineralocorticoid receptors in pituitary tumors causing Cushing’s disease and silent corticotroph tumors

**DOI:** 10.3389/fendo.2023.1124646

**Published:** 2023-03-29

**Authors:** Paulina Kober, Natalia Rusetska, Beata J. Mossakowska, Maria Maksymowicz, Monika Pękul, Grzegorz Zieliński, Andrzej Styk, Jacek Kunicki, Łukasz Działach, Przemysław Witek, Mateusz Bujko

**Affiliations:** ^1^ Department of Molecular and Translational Oncology, Maria Sklodowska-Curie National Research Institute of Oncology, Warsaw, Poland; ^2^ Department of Experimental Immunotherapy, Maria Sklodowska-Curie National Research Institute of Oncology, Warsaw, Poland; ^3^ Department of Cancer Pathomorphology, Maria Sklodowska-Curie National Research Institute of Oncology, Warsaw, Poland; ^4^ Department of Neurosurgery, Military Institute of Medicine, Warsaw, Poland; ^5^ Department of Neurosurgery, Maria Sklodowska-Curie National Research Institute of Oncology, Warsaw, Poland; ^6^ Department of Internal Medicine, Endocrinology and Diabetes, Medical University of Warsaw, Warsaw, Poland

**Keywords:** pituitary neuroendocrine tumor (PitNET), Cushing’s disease (CD), silent corticotroph adenoma, *NR3C1*, *NR3C2*, hypothalamic - pituitary - adrenal axis, glucocorticoid receptor, mineralocorticoid receptor

## Abstract

**Objective:**

Pituitary neuroendocrine corticotroph tumors commonly cause Cushing’s disease (CD) that results from increased adrenocorticotropic hormone (ACTH) secretion by the pituitary tumor and consequent increase of cortisol levels in blood. However, in some patients, corticotroph tumors remain clinically non-functioning. Cortisol secretion is regulated by the hypothalamic–pituitary–adrenal axis and includes a negative feedback between cortisol and ACTH secretion. Glucocorticoids reduce ACTH level both by hypothalamic regulation and acting on corticotrophs *via* glucocorticoid (GR) and mineralocorticoid (MR) receptors. The aim of the study was to determine the role of GR and MR expression at mRNA and protein levels in both functioning and silent corticotroph tumors.

**Methods:**

Ninety-five patients were enrolled, including 70 with CD and 25 with silent corticotroph tumors. Gene expression levels of *NR3C1* and *NR3C2* coding for GR and MR, respectively, were determined with qRT-PCR in the two tumor types. GR and MR protein abundance was assessed with immunohistochemistry.

**Results:**

Both GR and MR were expressed in corticotroph tumors. Correlation between *NR3C1* and *NR3C2* expression levels was observed. *NR3C1* expression was higher in silent than in functioning tumors. In CD patients *NR3C1* and *NR3C2* levels were negatively correlated with morning plasma ACTH levels and tumor size. Higher *NR3C2* was confirmed in patients with remission after surgery and in densely granulated tumors. Expression of both genes and GR protein was higher in *USP8*-mutated tumors. Similar relationship between *USP8* mutations and expression levels were observed in analysis of silent tumors that also revealed a negative correlation between GR and tumor size and higher *NR3C1* expression in densely granulated tumors.

**Conclusions:**

Although the associations between gene/protein expression and patients clinical features are not strong, they consistently show an evident trend in which higher receptor expression corresponds to more favorable clinical characteristics.

## 1 Introduction

Corticotroph pituitary neuroendocrine tumors (corticotroph PitNETs) are the subtype of tumors of pituitary gland (1). They develop as a result of neoplastic transformation of corticotropic cells of the anterior pituitary (1). Most of corticotroph PitNETs cause Cushing’s disease (CD) which is characterized by a set of symptoms caused by increased ACTH secretion and consequent serum cortisol excess. Approximately 20% of corticotroph tumors are clinically hormonally inactive (silent), i.e. do not cause rise in cortisol in patients’ circulation (2). The reason why a proportion of ACTH-secreting tumors remain clinically silent and do not cause Cushing’s disease remains unclear. Intriguingly, the molecular profiles of these two groups of corticotroph PitNETs are similar (3, 4).

Hypothalamic–pituitary–adrenal (HPA) axis is a neurohormonal system that provides a negative feedback between pituitary and adrenal secretion (5). Glucocorticoids inhibit ACTH secretion by reducing the level of hypothalamic CRH hormone as well as by direct inhibition of pituitary corticotroph cells activity (5). Partial resistance to glucocorticoids, a hallmark of Cushing’s disease, results from the impairment in HPA axis regulation (6).

Glucocorticoids signal through two steroid hormone receptors: glucocorticoid receptor (GR) and mineralocorticoid receptor (MR) (5, 7). Both receptors are intracellular proteins that upon ligand-dependent activation translocate to the nucleus, where they exert their role of transcription factors and regulate the expression of glucocorticoid-responsive genes. GR and MR have sequence homology and high similarity in DNA binding domains that are responsible for receptor interaction with glucocorticoid response elements (GREs) in DNA (5, 7). The receptors directly regulate the transcription of target genes and/or influence the expression indirectly by interacting with other transcription factors (5, 7, 8). In cells expressing both receptors, MR and GR bind GREs as homo- or heterodimers and the role of dimer composition is not fully understood (7).

The role of corticosteroid receptors in pituitary corticotroph tumors is relatively poorly understood (6, 9). In CD-causing tumors, loss of heterozygosity (LOH) in the *NR3C1* (encoding GR) locus was observed (10). More recently, somatic point mutations in *NR3C1* were found in 6.4% of patients with CD (9). *NR3C1* expression may be also downregulated by hsa-miR-124-3p miRNA in pituitary corticotroph tumors (11). Considering the growing interest in the role of GR and nearly no information on the role of MR in corticotroph PitNETs, we aimed to investigate the expression levels of both receptors in a relatively large cohort of functioning and silent corticotroph tumors.

## 2 Materials and methods

### 2.1 Patients’ characteristics

The study included 95 patients with corticotroph tumors of which 70 suffered from CD and 25 present with silent corticotroph PitNETs (silent corticotroph adenomas, SCAs). CD patients had clear clinical signs and symptoms of hypercortisolism verified according to biochemical criteria: increased urinary free cortisol (UFC) in three 24h urine collections; disturbed cortisol circadian rhythm, increased serum cortisol levels accompanied by increased or not suppressed plasma ACTH levels at 8.00; no suppression of serum cortisol levels to <1.8 µg/dL during an overnight dexamethasone suppression test (1 mg at midnight). The pituitary etiology of disease was confirmed based on the measurement of serum cortisol levels or UFC suppression <50% with a high-dose dexamethasone suppression test (2 mg q.i.d. for 48 h) or a positive result of a corticotropin-releasing hormone stimulation test (100 mg i.v.) and magnetic resonance imaging of pituitary. Patients with SCA had no clinical or biochemical signs of hypercortisolism and showed normal levels of midnight cortisol and 24h UFC. ACTH levels were assessed using IRMA (ELSA-ACTH, CIS Bio International, Gif-sur-Yvette Cedex, France). Serum cortisol levels were determined by the Elecsys 2010 electrochemiluminescence immunoassay (Roche Diagnostics, Mannheim, Germany). UFC was assessed after extraction (liquid/liquid with dichloromethane) by electrochemiluminescence immunoassay (Elecsys 2010, Roche Diagnostics, Mannheim, Germany).

All CD-related and SCA tumor samples were ACTH-positive upon immunohistochemical staining (IHC) against pituitary hormones (ACTH, GH, PRL, TSH, FSH, LH, α-subunit) and revealed characteristic ultrastructural features of corticotroph tumors, as assessed with electron microscopy. Each SCA sample was T-PIT positive as determined in additional immunohistochemical staining. Evaluation of Ki-67 immunoreactivity score in tumor sample was performed for all the patients.

Status of hot-spot mutation in ubiquitin-specific peptidase 8 (*USP8*) and ubiquitin-specific peptidase 48 (*USP48*) genes was determined in entire patients cohort with a method described previously (12).

Patients characteristics are presented in **Table 1**. The study was approved by the local Ethics Committee of Maria Sklodowska-Curie National Research Institute of Oncology, Warsaw, Poland (approval no. number 44/2018). Each patient provided informed consent for the use of tissue samples for scientific purposes.

**Table 1:** Summary of clinical features of patients with Cushing’s disease and silent corticotroph tumors.

Clinical Feature	Cushing’s Disease	Silent Corticotroph Tumors	
**Number of patients**	n = 70	n=25	
**Sex** (ratio females/males)	59/11	11/14	p = 0.0003
**Age at surgery** (years; median (range))	43.5 (15-78)	46 (23-77)	p > 0.05
**Cortisol 08:00 h** (µg/dL; median (range))	25.10 (11.54-73.40)	15.78 (6.8-50.8)	p < 0.0001
**Cortisol 24:00 h** (µg/dL; median (range))	18.4 (6.83-42.80)	1.1 (0.3-10.6)	p < 0.0001
**ACTH 08:00 h** (pg/dL; median (range))	82.29 (31-390)	41.7 (12.5-74.9)	p < 0.0001
**UFC** (μg/24 h; median (range))	422.5 (163.9 - 1230)	97.4 (13.7-139)	p < 0.0001
**Tumor largest size** (mm; median (range))	12 (3 -62)	24.5 (15-35)	p < 0.0001
**Invasive tumor growth** (Knosp grade 0, 1, 2/3, 4)	49/21	20/5	p > 0.05
**Ultrastructure (electron microscopy)** (sparsely/densely granulated)	20/49*	15/10	p = 0.0082
**Ki-67 score** (≤3%/>3%)	12/58	2/23	p > 0.05

* One patient with Crooke cell histology was included.

### 2.2 Quantitative real-time RT-PCR

Total RNA from formalin-fixed and paraffin-embedded (FFPE) tumor tissues was isolated with RecoverAll™ Total Nucleic Acid Isolation Kit for FFPE [Thermo Fisher Scientific, Waltham, Massachusetts, USA], measured using NanoDrop 2000 [Thermo Fisher Scientific, Waltham, Massachusetts, USA] and stored at -70 °C. One microgram of total RNA was subjected to reverse transcription using Transcriptor First Strand cDNA Synthesis Kit (Roche Diagnostics). Power SYBR Green PCR Master Mix (Thermo Fisher Scientific) was used for qRT-PCR reaction, according to manufacturer’s recommendations. The reactions were run in a volume of 5 μL, containing 2.25 pmol of each primer, using 384-well format in 7900HT Fast Real-Time PCR System (Applied Biosystems). Each sample was amplified in triplicates. Delta Ct method was used to calculate relative expression levels of *NR3C1* and *NR3C2*, and *GAPDH* was used as a reference gene. The following oligonucleotides were used as PCR primers: NR3C1 forward 5’-TGCTCCTTCTGCGTTCACAA-3’, NR3C1 reverse 5’-CCATCAGTGAATATCAACTCTGGC-3’, NR3C2 forward 5’-GGGGATGAGGCTTCAGGATG-3’, NR3C2 reverse 5’-AGTTGTGTTGCCCTTCCACT-3’, GAPDH forward 5’-GAAGATGGTGATGGGATTTC-3’, GAPDH reverse 5’-GAGGTGAAGGTCGGAGTC-3’.

### 2.3 Immunohistochemistry

IHC was performed on 4-μm FFPE tissue sections using Envision Detection System (Dako, Glostrup, Denmark), according to manufacturer’s recommendations. Tissue sections were deparaffinized with xylene and subsequently rehydrated in a series of ethanol solutions of decreasing concentration. Target Retrieval Solution pH 9 (Dako) was used for heat-induced epitope retrieval in a 96°C water bath for 30 minutes. The samples were incubated for 1 h with the primary antibodies: anti-glucocorticoid receptor antibody (EPR19621, Abcam) and anti-mineralocorticoid receptor antibody (H10E4C9F, Abcam) in 1:100 and 1:200 dilutions, respectively. Color reaction product was developed with 3,3′-diaminobenzidine tetrahydrochloride (Dako) as a substrate. Hematoxylin counterstaining was applied. Analysis of nuclear immunohistochemical reactivity was performed by calculating H-score with a formula that combines information on both reaction intensity (scored from 0 to 3) and number of the cells with a given intensity (13). Scoring results were analyzed as continuous variables. Intensity of cytoplasmic reactivity was assessed in 4-grade scale (0-3), where 0 was considered as no expression, 1- low expression, 2 – moderate expression, 3 – high protein level and the results were analyzed as categorical variables.

### 2.4 Statistical analysis

Datasets of quantitative variables were tested for the normal distribution with Shapiro-Wilk test. Variables following normal distribution were analyzed with two-sided t-test whereas two-sided Mann–Whitney U-test was used when normal distribution was not determined. The Spearman correlation method was used for correlation analysis. Exact Fisher’s test was used for the analysis of proportions. Significance threshold of α = 0.05 was adopted. Data was analyzed using GraphPad Prism 6.07 (GraphPad Software, San Diego, California, USA).

## 3 Results

### 3.1 GR and MR expression in silent and functioning corticotroph tumors

**Figure 1:**
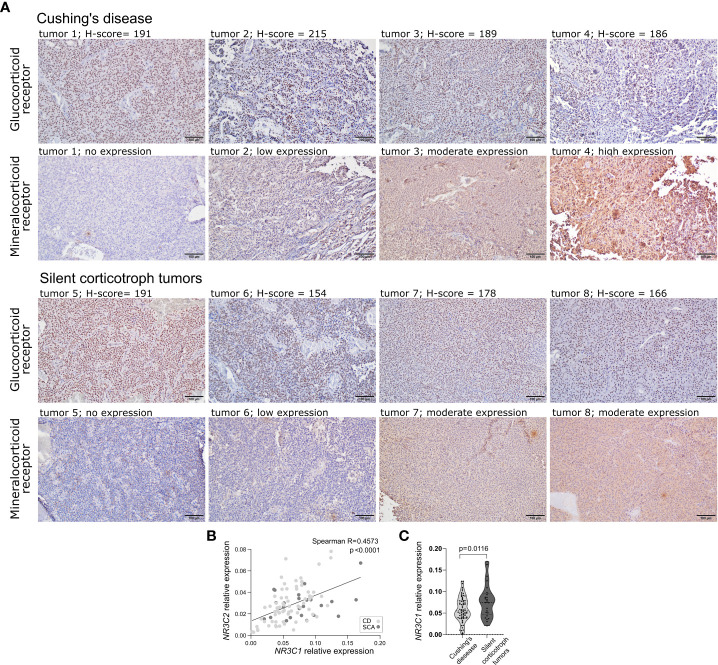
The expression of corticosteroid receptors in corticotroph tumors. **(A)** Representative examples of results of immunohistochemical staining against glucocorticoid receptor and mineralocorticoid receptor. **(B)** Difference in *NR3C1* expression in silent corticotroph pituitary tumors and functioning corticotroph tumors causing Cushing’s disease (CD). **(C)** The correlation between the expression of *NR3C1* and *NR3C2* in corticotroph tumors.

The expression levels of *NR3C1* and *NR3C2* were determined in 25 SCAs and 70 functioning corticotroph tumors with qRT-PCR. Protein GR and MR expression was assessed based on immunohistochemical staining in the same tumor samples as used for qRT-PCR. Predominant nuclear expression was observed for GR, and immunoreactivity was quantified by counting positive nuclei according to H-score formula. For MR, cytoplasmic immunoreactivity was observed with additional slight nuclear staining in few samples only. Due to difficulties with counting the individual MR-positive cells, a simplified score was applied which categorized MR expression in tissue samples as negative, weak, moderate and high. Five tumor samples were lacking MR expression, 35 had weak, 51 moderate and 1 high MR expression. Two CD patients that were included in qRT-PCR analysis were excluded from immunohistochemical assessment due to low quality of tissue samples. Intracellular localization of each protein was highly similar across the samples. Representative examples of results of immunohistochemical staining are presented in **Figure 1A**.

We did not observe clear relationship between mRNA and protein levels. No correlation was observed between *NR3C1* and GR immunoreactivity, quantified using H-score formula (Spearman R = 0.03801; p = 0.716). No difference in *NR3C2* expression between samples with distinct MR immunoreactivity (no expression, weak, moderate, strong expression) was found (p = 0.9625, Kruskal-Wallis test). Clear correlation was found between *NR3C1* and *NR3C2* expression (Spearman R = 0.4573, p < 0.0001) (**Figure 1B**). However, no difference in GR immunoreactivity score between MR expression categories was observed indicating lack of relationship between the expression at the protein levels.

When comparing silent and functioning corticotroph tumors a significantly higher expression of *NR3C1* was observed in SCAs than in tumors from CD patients (expression fold change (FC) = 1.36; p = 0.0166) (**Figure 1C**). No difference in *NR3C2* expression, GR or MR immunoreactivity was found.

### 3.2 Relationship between the expression of corticosteroid receptors and clinical parameters in patients with functioning and silent corticotroph tumors

SCA and CD differ in terms of diagnosis and patients’ treatment, thus the roles of the expression of corticosteroid receptors in the two patients groups were assessed separately.

We analyzed the relationship between the expression of *NR3C1*, *NR3C2*, GR and MR with clinical and pathomorphological features: morning ACTH level, morning cortisol level, midnight cortisol level, 24h UFC, clinical remission in CD patients, tumor size, invasive growth status, *USP8*/*USP48* mutation status, granulation pattern (sparsely granulated (SG) *vs* densely granulated (DG)) and Ki-67 score.

**Figure 2:**
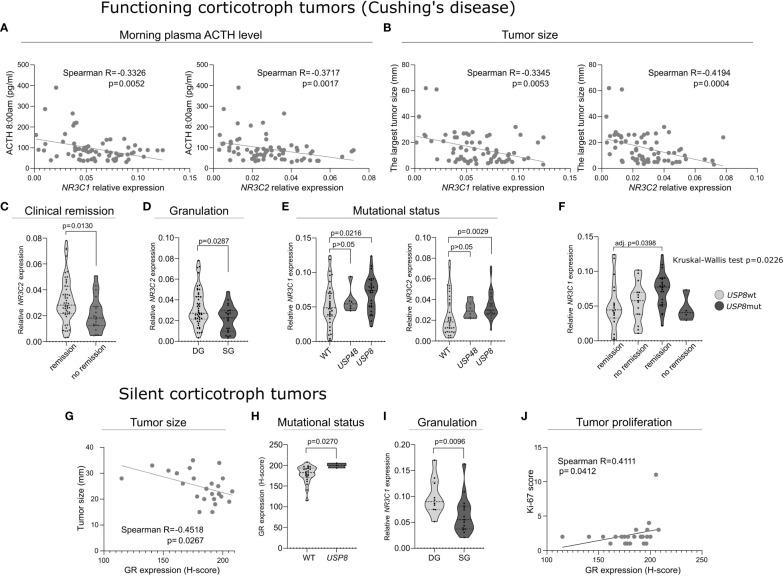
The expression of corticosteroid receptors’ genes and proteins in functioning and silent corticotroph tumors. **(A)** The correlation between morning plasma ACTH level and the expression of *NR3C1* and *NR3C2* expression in tumor tissue in Cushing’s disease patients. **(B)** The correlation between tumor size and the expression of *NR3C1* and *NR3C2* in Cushing’s disease patients. **(C)** Difference in *NR3C2* expression in tumors of Cushing’s disease patients with clinical remission after surgery and those without remission. **(D)** Difference in *NR3C2* expression between densely granulated (DG) and sparsely granulated (SG) functioning corticotroph pituitary tumors. **(E)** Difference in the expression of *NR3C1* and *NR3C2* in tumors with mutations in deubiquitinase-encoding genes (*USP8* and *USP48*) and wild type functioning corticotroph pituitary tumors. **(F)** The expression of *NR3C1* in patients with and without remission after surgery with respect to *USP8* mutation status. **(G)** Correlation between the expression of glucocorticoid receptor (GR) and tumor size in silent corticotroph tumors. **(H)** GR expression in SCAs with and without *USP8* mutation. **(I)** Difference in *NR3C2* expression between densely granulated (DG) and sparsely granulated (SG) silent corticotroph pituitary tumors. **(J)** Correlation between GR expression and Ki-67 immunoreactivity score in silent tumors.

The analysis of CD patients data revealed that both *NR3C1* and *NR3C2* expression levels were negatively correlated with morning plasma ACTH levels (Spearman R = -0.3326; p = 0.0052 and R= -0.3717; p = 0.0017, respectively) and tumor size (Spearman R = -0.3345; p = 0.0053 and R = -0.4194; p = 0.0004, respectively) (**Figures 2A, B**). Patients with clinical remission after surgery had higher *NR3C2* mRNA level (FC = 1.545; p = 0.013) (**Figure 2C**). Slightly higher *NR3C2* expression level was also observed in DG tumors as compared to SG ones (FC = 1.2; p = 0.0295) (**Figure 2D**). We did not observe relationship between expression of the receptors and cortisol level, 24h UFC, invasive growth status and Ki-67 score. All the results are shown in details in **Supplementary Table 1**.

Mutations in genes encoding protein deubiquitinases *USP8* and *USP48* were determined in tumor tissue in the entire cohort of patients. *USP8* mutations were found in 28/70 (40%) of patients with CD. The identified variants were: p.P720R (n = 13), p.S718SP (n = 5), pP720Q (n = 2), and in-frame deletion at position S719 (n = 8). *USP48* missense mutations p.M415I were observed in 5 (7.14%) CD patients. *USP48* and *USP8* mutations were mutually exclusive. *USP8* mutations were identified in 3 patients with silent tumors, that we already reported previously (4). They were p.S718SP (n=1) and in-frame deletion at position S719 (n = 2). No *USP48* mutations were detected in SCA patients. CD patients with *USP8* mutations had significantly higher expression of both *NR3C1* and *NR3C2* (FC = 1.508; p = 0.0216 and FC = 1.626; p = 0.0029, respectively) (**Figure 2E**).

Published data indicate that tumors with *USP8* mutations and *USP8*-wild type tumors have distinct molecular profiles (3, 4). Since different expression of *NR3C1* and *NR3C2* was found in mutated and non-mutated tumors, we additionally ran analysis of relationship between the expression of each gene and clinical features in subgroups of tumors with *USP8* mutation and wild type tumors (without *USP8* and *USP48* mutations). This showed negative correlation between *NR3C2* expression and ACTH level, in analysis of both groups of mutated and wild type tumors (Spearman R = -0.406; p = 0.0356 and R = -0.4044; p = 0.0196, respectively). Additionally, analysis performed in the subgroup of patients with *USP8* mutations showed higher *NR3C1* expression in patients with clinical remission after surgery than in patients without remission. Similar result was not found in the group of patients with non-mutated tumors. Subsequent comparison of 4 groups of CD patients clustered according to *USP8* mutation status and clinical remission status, with *post hoc* analysis showed that *NR3C1* expression is significantly higher in *USP8*-mutated patients with clinical remission after surgery than in non-mutated patients with clinical remission (adj. p = 0.0398) (**Figure 2F**).

The analysis of silent corticotroph tumors showed a negative correlation between GR immunoreactivity score and tumor size (Spearman R = -0.4518; p = 0.0267) (**Figure 2G**), however, it was not observed for the relative *NR3C1* and *NR3C2* expression levels in this patients’ group. Lower immunoreactivity score was also found in *USP8*wt SCAs compared to in *USP8-*mutated tumors (H-score 200 vs 182.4, respectively; p = 0.0279) (**Figure 2H**). Higher relative expression of *NR3C1* and *NR3C2* in *USP8*-mutated tumors was also observed in the analyzed SCAs, however, it did not cross significance threshold (FC = 1.47; p = 0.0587 and FC = 1.80; p = 0.0587, respectively). Of note, in these analyses only 3 *USP8*-mutated samples were available. Densely granulated SCAs had higher *NR3C1* expression level than sparsely granulated ones (FC = 1.63; p = 0.0116) (**Figure 2I**), but no difference in *NR3C2* expression or corticosteroid receptors protein expression was found. We also found a significant correlation between GR expression and Ki-67 immunoreactivity score treated as continuous variable (Spearman R = 0.4111; p = 0.0412) (**Figure 2J**). However it has to be noted that our study group contains only 2 SCA patients with Ki-67>3% what limits the quality of the analysis.

In silent corticotroph tumors we did not observe relationship between *NR3C1*, *NR3C2*, GR or MR expression and clinical parameters (morning ACTH level, morning cortisol level, midnight cortisol level, 24h UFC, invasive growth status). All the results on silent corticotroph tumors are presented in details in **Supplementary Table 2**.

## 4 Discussion

Corticotroph PitNETs can be divided into functioning and silent tumors. They substantially differ in tumor clinical manifestation and results of biochemical tests. These differences are the basis for discriminating functioning corticotroph, CD-causing tumors and silent tumors. Functioning corticotroph PitNETs occur more frequently in women, while this sex-related prevalence is not observed in SCAs (14–16). Concordant difference in proportions of males and females in groups of CD and SCA patients was also observed in our patients cohort. Functioning corticotroph PitNETs are commonly small in size and are diagnosed due to excessive hormone secretion while SCAs are larger and commonly diagnosed following neurological symptoms caused by tumor volume. Accordingly, in our patients cohort SCAs are significantly larger than functioning tumors. In general, SCAs are considered as more aggressive tumors having higher rate of invasive growth and commonly they show sparse granulation pattern that is related to worse prognosis (17–19). In our study SG tumors were more prevalent in SCA patients while DG tumors were more frequent among CD patients that is also in line with previous reports (19). However, in our patient group we did not observe a difference in invasive growth status determined with Knosp’s grading or proliferation rate assessed based on Ki-67 staining. Majority of the tumors in each group were non-invasive ones with Ki-67 score below 3%. Low rate of invasive tumors may indicate that our group of SCA patients is not fully representative that should be taken into account with generalization of our results.

Glucocorticoids play a pivotal role in the regulation of hypothalamic–pituitary–adrenal (HPA) axis in a negative feedback mechanism. They inhibit ACTH secretion both indirectly *via* hypothalamus (by reducing CRH secretion) and directly acting on pituitary corticotroph cells (5). Glucocorticoids can be bound by two intracellular receptors GR and MR that regulate transcription of target genes (5, 7). It was shown in corticotroph cells that upon stimulation with glucocorticoids GR regulates *POMC* expression though various mechanisms including binding of GR to negative GREs (nGREs) in gene promoter (20), chromatin remodeling (21) or interaction with other nuclear receptor (NR4A1) involved in *POMC* regulation (22). Upon stimulation, GR may also act in nongenomic way by regulating membrane permeability for calcium influx (23). The function of GR and MR receptors in regulation of corticotroph cells activity suggests that they may play an important role in pituitary corticotroph tumors. According to our best knowledge, only few studies on limited number of patients were performed to address the role of GR expression and nearly no research on the role on MR expression in corticotroph PitNETs were published.

In our previous study comparing silent and functioning pituitary corticotroph tumors we found a difference in the expression level of genes encoding GR and MR (*NR3C1* and *NR3C2*, respectively) (11). In the current investigation we included a notably higher number of patients to validate previous results. The obtained data confirmed a higher *NR3C1* expression in silent tumors as compared to tumors causing CD, but we did not observe the difference in *NR3C2* level. Our results show that, although statistically significant, the difference is slight. This observation supports our previous suggestion that higher *NR3C1* expression has rather modulatory contribution to silent nature of SCAs than a strong causative role. The general model of GR activity indicates that its higher expression may directly decrease ACTH secretion (5) and contribute to non-secreting nature of SCAs. Importantly, secretory activity of corticotroph cells is regulated in a complex way. Previous studies comparing silent and functioning corticotroph tumors showed that they differ in several elements of this regulation including *POMC* expression, prohormone convertases level, dopamine or somatostatin receptor level and expression of specific ion channel (4, 24–28). We suppose that changes of few regulatory elements can occur simultaneously with additive effect on functioning of corticotroph tumors and GR/MR expression can be one of these changes. The possibly distinct expression of *NR3C1* in functioning and silent corticotroph tumors was previously investigated in smaller series of patients but no significant difference was found in these studies (27, 28). This was probably due to lower patients’ numbers, i.e. n = 50 (36 CD, 14 SCA) and n = 20 (12 CD, 8 SCA), respectively and a small difference in gene expression levels. Previous analysis of GR protein expression in SCA and CD-causing tumors also did not reveal difference between the tumors of diverse functional status (29).

The change of functional status of corticotroph tumor can be observed in some patients. Cases of spontaneous conversion of silent adenoma into CD-causing corticotroph tumor as well as functioning to non-functioning tumor were reported (30, 31). Periodic changes of the secretory activity of the tumor are also considered as probable cause of cyclic Cushing's syndrome in patient with ACTH-related hypercortisolemia who experience temporal switch between hypercortisolemia and normocortisolemia status (32). The observed different NR3C1 expression between silent and functioning corticotroph tumors suggest that changes in GR expression may also contribute to temporary changes of tumor activity. Obviously, our speculation require further investigation. Recently, the role of GR in CD pathogenesis attracted the attention since new somatic point mutations in *NR3C1* gene in functioning corticotroph tumors were identified (33). It has been estimated that these mutations occur in 6.2% of CD patients (34) although the functional role of most of identified somatic variants is unclear and was not investigated *in vitro*. Three *NR3C1* mutations that were found in study by Miao et al. were shown to produce truncated GR protein or to reduce GR expression level (35).

The role of GR expression in patients with Cushing’s disease was not investigated extensively and the results of only a few studies were published. In general, these studies did not reveal any relationship of *NR3C1* gene expression level and patients’ clinical features (27, 28, 36, 37) or GR protein expression with patients’ characteristics (29). One study indicated the relationship between low *NR3C1* expression and the response to high dose dexamethasone suppression test, however, only six functioning corticotroph tumors were used in this evaluation (38). Of note, most of these previous studies included relatively low number of CD patients: n=17 (36), n=6 (38), n=12 (27), n=8 (29), n=36 (28) with only one study including more than 50 persons (37). Interestingly, although GR and MR are both involved in the response to glucocorticoids, to our knowledge, there is no data on *NR3C2* or MR protein expression in CD-causing tumor available to date. Initially, the expression of MR was considered more tissue-specific as compared to GR, with highest levels in kidney and adipose tissue but its expression was identified in multiple human tissues (39). According to publicly available databases on tissue specific gene/protein expression in human MR is widely expressed in most tissue types/organs including normal pituitary gland (Geno-type-Tissue Expression project (https://gtexportal.org) or Protein atlas (https://www.proteinatlas.org), accessed on November, 2022). The expression of both MR and GR was documented in PVN neurons, adipocytes, osteoblasts, immune cells and kidney cells (40–44).

In our study we found some interesting relationships between *NR3C1* and *NR3C2* expression and clinical/histological features in CD patients. The expression levels of both receptors were negatively correlated with plasma ACTH level and tumor size. The expression of *NR3C2* was also higher in patients with post-surgery clinical remission than in patients who did not experience biochemical remission after surgery and in patients with DG tumors as compared to SG tumors. DG histology is considered as related to favorable clinical outcome (19). Expression levels of both genes were also higher in tumors with *USP8* mutations, which are the most common driver mutations in corticotroph PitNETs. These mutations are generally related to smaller tumor size and less aggressive growth (28, 34) *USP8*-mutatated and wild type tumors are also characterized by different biological features (34). The expression of *NR3C1* turned out to be the highest in *USP8*-mutated tumors from patients with clinical remission after surgery indicating that CD patients with this mutation and high *NR3C1* level have the best chance for clinical remission.

The relationships between the expression levels of GR/MR-encoding genes and clinical parameters revealed in our study were not strong but statistically significant. This allows to cautiously generalize the results of this study. They clearly show a trend in which higher receptor expression corresponds to more favorable clinical profile in patients, including lower plasma ACTH level, smaller tumor size, clinical remission after surgery and favorable features of sparsely granulated, *USP8*-mutated tumors.

Due to a weak relationship with patients characteristics it is difficult to indicate any clinical usefulness of testing *NR3C1* or *NR3C2* expression but our data support the clinically relevant roles of the receptors expression levels in functioning corticotroph pituitary tumors.

Probably the clinically relevant results were not observed in previous studies on *NR3C1* expression in CD due to lower number of patient and the fact that the observed relationship is not strong. We suppose that the small difference in gene expression affects the general lack of the finding of the significant associations in our GR and MR protein level analysis. While qRT-PCR results are numerical data by definition, immunohistochemistry is a qualitative method and based on subjective microscopic observations, thus, it is generally less accurate. In our setting, GR immunostaining evaluation produced a relatively small range of immunoreactivity scores and similarly for MR we observed mainly low or moderate immunoreactivity. This is probably also the reason why we did not observe a correlation between qRT-PCR results and protein quantification in our study, and any difference in GR expression when comparing SCAs and functioning tumors.

Targeting GR with receptor antagonist is one of the main pharmacological options in Cushing’s syndrome (CS) aimed to reduce cortisol-related complications (9). The efficacy of two GR modulators mifepristone and relacorilant were already tested and subjected to phase III clinical trials (9). In case of ACTH-related hypercortisolemia this therapeutical approach may provide both therapeutical benefit and adverse effect due to direct effect on corticotroph tumor. Blocking the receptors in tumor may decline the inhibitory effect of GR on ACTH secretion i.e. direct negative adrenal-pituitary feedback. In fact, the use of mifepristone in CD was shown to result in more than 2-fold increase of ACTH secretion in patients (45). More promising preliminary results were obtained in the evaluation of relacorilant (46), but phase III clinical trials are still in progress. Our observation of the negative correlation between the tumor expression of corticosteroid receptor genes and plasma ACTH level suggests that lower receptors levels contribute to less effective negative regulation and possibly could affect lower adverse effect of GR antagonist on direct negative adrenal-pituitary feedback. We suppose that evaluation of expression status of GR/MR receptors (at both gene and protein level) may provide some predictive value in CD patients treated with GR modulators. Perhaps, patients with low expression could benefit more from the treatment. This would be important because our data indicate that these patients are of worse prognosis. This issue requires further specific research. The roles of GR and MR are considered cell-type specific. These receptors may play distinct, even opposite functions such as in immune cells where MR mediates proinflammatory effects, while GR mediates immune suppression (47). Both receptors have pivotal roles in the regulation of HPA axis and reducing activity of pituitary corticotrophs. MR is considered to be ligand-activated throughout the entire circadian cycle, including periods of low circulating glucocorticoid concentrations (inactive phase), when GR receptor is inactive. In turn, GR receptor is activated at high glucocorticoid circadian phase (active phase) or during a stress response (5). These different roles probably result from notably higher affinity of MR to glucocorticosteroids and longer substrate binding time as compared to GR (5). However, more recent data indicate more dynamic activity of MR and circadian differences in its occupancy at particular target DNA loci, as well as cooperative action of both receptors forming MR : GR heterodimers (48, 49). Most of the published data on MR and GR role in regulation of HPA-axis concern their role in hypothalamic neurons while the mechanism of their interaction in corticotroph cells was not determined. Importantly, our observations of co-expression of *NR3C1* and *NR3C2* in corticotroph PitNETs and negative correlation between the expression of each gene and morning plasma ACTH level suggest that in pituitary corticotroph cells the receptors may synergistically regulate secretory activities of hypothalamic neurons. This hypothesis needs verification in functional research regarding the role of MR in pituitary corticotrophs and cooperation between GR and MR in this type of cells.

## 5 Conclusions

GR and MR are both expressed in corticotroph tumors and there is a correlation between mRNA levels of genes encoding these receptors. *NR3C1* expression level is higher in silent than in functioning corticotroph tumors. In CD patients there is an evident trend towards more favorable clinical characteristics in tumors with higher receptors’ expression.

## Data availability statement

The raw data supporting the conclusions of this article will be made available by the authors, without undue reservation.

## Ethics statement

The studies involving human participants were reviewed and approved by Ethics Committee of Maria Sklodowska-Curie National Research Institute of Oncology, Warsaw, Poland. The patients/participants provided their written informed consent to participate in this study.

## Author contributions

PK, MB and MM contributed to conception and design. PK, NR, BM and MP contributed to investigation and acquiring laboratory results. GZ, JK, LD, PW and MM contributed to investigation and acquiring of resources (tissue samples and data for the analysis). PK, MM, MB contributed to visualization. PK, MB contributed to data analysis. PK, MB wrote the first draft of the manuscript. PW, MM contributed to writing—review and editing the manuscript. All authors contributed to the article and approved the submitted version.
