# Supplementary material for: The expression of glucocorticoid and mineralocorticoid receptors in pituitary tumors causing Cushing’s disease and silent corticotroph tumors
Source: Front Endocrinol (Lausanne). 2023 Mar 29;14:1124646. doi: 10.3389/fendo.2023.1124646 (PMC10090509; doi:10.3389/fendo.2023.1124646)
Supplement: Supplementary file 1 [file Table_1.docx]

Supplementary Material

The expression of glucocorticoid and mineralocorticoid receptors in corticotroph pituitary neuroendocrine tumors

Paulina Kober. Natalia Rusetska. Beata J. Mossakowska. Maria Maksymowicz. Monika Pękul^2^. Grzegorz Zieliński. Andrzej Styk. Jacek Kunicki. x x . Przemysław Witek. and Mateusz Bujko^*^

*** Correspondence:** Corresponding Author: mateusz.bujko@pib-nio.pl

# Supplementary Tables

|  | cortisol 08.00 (ug/dl) | | ACTH 08.00 (pg/dl) | | 24h UFC (4.3-176 ug/24h) | | Midnight cortisol (ug/dl) | | Clinical remission | | The largest tumor size | | Ki67 score | | invasive growth | | SG vs DG | | *USP8*mut vs wt | |
| --- | --- | --- | --- | --- | --- | --- | --- | --- | --- | --- | --- | --- | --- | --- | --- | --- | --- | --- | --- | --- |
|  | Spearman R | p-value | Spearman R | p-value | Spearman R | p-value | Spearman R | p-value | FC | p-value | Spearman R | p-value | Spearman R | p-value | FC | p-value | FC | p-value | FC | p-value |
| *NR3C1* expression* | -0.067 | 0.579 | **-0.333** | **0.005** | -0.141 | 0.264 | -0.130 | 0.297 | 1.07 | 0.1708 | **-0.335** | **0.005** | 0.1180 | 0.3307 | 1.21 | 0.216 | 1.17 | 0.133 | **1.50** | **0.022** |
| *NR3C2* expression* | -0.140 | 0.243 | **-0.372** | **0.002** | -0.1066 | 0.398 | 0.009624 | 0.939 | **1.54** | **0.013** | **-0.419** | **0.0004** | -0.02148 | 0.8599 | 1.10 | 0.173 | **1.20** | **0.029** | **1.63** | **0.0029** |
| GR expression (H-score)* | 0..147 | 0..156 | 0..014 | 0..893 | 0..109 | 0..34 | 0..162 | 0..151 | 1..02 | 0..223 | -0..12 | 0..341 | 0.08049 | 0.5077 | 1.02 | 0.120 | 1.01 | 0.745 | **1..02** | **0..0496** |
|  | FC | p-value | FC | p-value | FC | p-value | FC | p-value | odds ratio | p-value | FC | p-value | FC | p-value | odds ratio | p-value | odds ratio | p-value | odds ratio | p-value |
| MR expression (lacking/weak vs moderate/strong expression)** | 1..09 | 0..408 | 1..13 | 0..804 | 1..40 | 0..388 | 1..01 | 0..984 | 0..43 | 0..177 | 1..14 | 0..995 | 1.04 | 0.103 | 1..44 | 0..568 | 0..53 | 0..406 | 1..25 | 0..793 |

**Supplementary Table 1.** The results of analysis of the relationship between the expression of corticosteroid receptors in functioning corticotroph tumors and clinical features in patients suffering from Cushing’s disease

FC- fold change; * indicates that Spearman correlation was used for analyzing cortisol. ACTH levels. 24h UFC and tumor size. while Mann-Whitney test was used for the analysis of invasive growth. ultrastructural granulation pattern and mutational status; ** indicates that Mann-Whitney test was used for analyzing cortisol. ACTH levels. 24h UFC and tumor size. while Fisher’s exact test was used for the analysis of the proportions in tumors with diverse clinical remission. invasive growth. ultrastructural granulation pattern and *USP8* mutation status

**Supplementary Table 2.** The results of analysis of the relationship between the expression of corticosteroid receptors in silent corticotroph tumors and patients’ clinical features

|  | cortisol 08.00 (ug/dl) | | ACTH 08.00 (pg/dl) | | 24h UFC (4.3-176 ug/24h) | | Midnight cortisol (ug/dl) | | The largest tumor size | | Ki67 score | | invasive growth | | SG vs DG | | *USP8*mut vs wt | |
| --- | --- | --- | --- | --- | --- | --- | --- | --- | --- | --- | --- | --- | --- | --- | --- | --- | --- | --- |
|  | Spearman R | p-value | Spearman R | p-value | Spearman R | p-value | Spearman R | p-value | Spearman R | p-value | Spearman R | p-value | FC | p-value | FC | p-value | FC | p-value |
| *NR3C1* expression* | 0.337 | 0.099 | 0.218 | 0.306 | 0.064 | 0.808 | 0.084 | 0.733 | -0.082 | 0.704 | 0.124 | 0.554 | 1.09 | 0.2863 | **1.63** | **0.012** | 1.47 | 0.059 |
| *NR3C2* expression* | -0.060 | 0.777 | -0.306 | 0.146 | 0.380 | 0.132 | -0.238 | 0.326 | -0.087 | 0.687 | 0.041 | 0.949 | 1.12 | 0.9433 | 1.14 | 0.454 | 1.80 | 0.058 |
| GR expression (H-score)* | -0.313 | 0.127 | -0.383 | 0.065 | 0.181 | 0.486 | -0.012 | 0.960 | **-0.452** | **0.027** | **0.411** | **0.041** | 1.09 | 0.055 | 1.01 | 0.677 | **1.10** | **0.028** |
|  | FC | p-value | FC | p-value | FC | p-value | FC | p-value | FC | p-value | FC | p-value | FC | p-value | odds ratio | p-value | odds ratio | p-value |
| MR expression (lacking/weak vs moderate/strong expression)** | 1.20 | 0.703 | 1.15 | 0.817 | 1.32 | 0.963 | 1.55 | 0.345 | 1.13 | 0.908 | 1.69 | 0.1135 | 1.67 | 0.615 | 1.71 | 0.513 | 0.5 | 0.588 |

FC- fold change; * indicates that Spearman correlation was used for analyzing cortisol. ACTH levels. 24h UFC and tumor size. while Mann-Whitney test were used for the analysis of invasive growth. ultrastructural granulation pattern and mutational status; ** indicates that Mann-Whitney test was used for analyzing cortisol. ACTH levels. 24h UFC and tumor size. while Fisher’s exact test was used for the analysis of the proportions in tumors with diverse invasive growth. ultrastructural granulation pattern and *USP8* mutation status;
